# The unfolded protein response is required to maintain the integrity of the endoplasmic reticulum, prevent oxidative stress and preserve differentiation in *β*-cells

**DOI:** 10.1111/j.1463-1326.2010.01281.x

**Published:** 2010-10-01

**Authors:** R J Kaufman, S H Back, B Song, J Han, J Hassler

**Affiliations:** 1 Howard Hughes Medical Institute, University of Michigan Medical CenterAnn Arbor, MI, USA; 2 Department of Biological Chemistry, University of Michigan Medical CenterAnn Arbor, MI, USA; 3 Department of Internal Medicine, University of Michigan Medical CenterAnn Arbor, MI, USA

**Keywords:** antioxidant, apoptosis, CHOP, eukaryotic initiation factor 2, mitochondria, PERK, protein folding, translation

## Abstract

Diabetes is an epidemic of worldwide proportions caused by *β*-cell failure. Nutrient fluctuations and insulin resistance drive *β*-cells to synthesize insulin beyond their capacity for protein folding and secretion and thereby activate the unfolded protein response (UPR), an adaptive signalling pathway to promote cell survival upon accumulation of unfolded protein in the endoplasmic reticulum (ER). Protein kinase-like endoplasmic reticulum kinase (PERK) signals one component of the UPR through phosphorylation of eukaryotic initiation factor 2 on the *α*-subunit (eIF2*α*) to attenuate protein synthesis, thereby reducing the biosynthetic burden. *β*-Cells uniquely require PERK-mediated phosphorylation of eIF2*α* to preserve cell function. Unabated protein synthesis in *β*-cells is sufficient to initiate a cascade of events, including oxidative stress, that are characteristic of *β*-cell failure observed in type 2 diabetes. In contrast to acute adaptive UPR activation, chronic activation increases expression of the proapoptotic transcription factor CAAT/enhancer-binding protein homologous protein (CHOP). *Chop* deletion in insulin-resistant mice profoundly increases *β*-cell mass and prevents *β*-cell failure to forestall the progression of diabetes. The findings suggest an unprecedented link by which protein synthesis and/or misfolding in the ER causes oxidative stress and should encourage the development of novel strategies to treat diabetes.

## Introduction

Type 2 diabetes is an epidemic in which tissues, such as muscle, fat and liver, become resistant to insulin and the pancreas fails to compensate adequately [1]. Insulin deficiency or resistance causes hyperglycaemia and hyperlipidaemia characteristic of the diabetic state. Unfortunately, little is known about how the *β*-cell responds to elevated blood glucose to increase insulin transcription, translation and secretion. When the *β*-cell compensates for insulin resistance, it must restructure the secretory apparatus to support high-level insulin production. Recent studies suggest that the ability of *β*-cells to compensate for hyperglycaemia-driven increases in insulin production requires an intracellular signalling pathway, the unfolded protein response (UPR) that emanates from the endoplasmic reticulum (ER).

## The Endoplasmic Reticulum: Protein Folding, Quality Control and ERAD

The ER is the site where proteins destined for the cell surface and endomembrane system enter the secretory pathway. Approximately one third of all proteins translocate across the ER membrane, where they fold into their proper three-dimensional structures and are subject to glycosylation, hydroxylation, lipidation and disulphide bond formation [2]–[4]. The ER contains an extremely high Ca^2+^ concentration and is occupied by chaperone proteins, catalysts of protein folding and enzymatic machinery for post-translational modifications [5]. Only properly folded proteins exit the ER to the Golgi compartment for further processing before trafficking to their final destination. The mechanisms for this exquisitely sensitive and specific quality control are under active investigation, but are not yet well defined. Retention of unfolded proteins in the ER lumen occurs through interaction with ER-resident peptide-binding proteins, such as BiP (immunoglobulin heavy chain-binding protein) and GRP94 (glucose-regulated protein). ER-associated protein degradation (ERAD) is the process whereby misfolded proteins are recognized in the ER, targeted to a channel within the ER membrane, extracted from the ER membrane and delivered to the proteasome [6]. At a minimum, quality control is mediated through the processing of *N*-linked glycans that are primarily recognized by lectin-binding proteins in the ER lumen [7]. These lectins direct refolding (e.g. CNX, CRT), anterograde transport (e.g. LMAN1) or ERAD (e.g. EDEMs, OS-9, XPT3-B) [8],[9].

**Figure 1:**
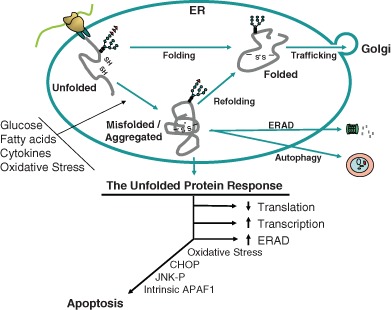
Pathways of protein misfolding that lead to cell death. Nascent unfolded polypeptides enter the endoplasmic reticulum (ER) and interact with chaperones and catalysts of protein folding to mature into compact, thermodynamically favourable structures. Failure of this process results in persistence of misfolded polypeptide–chaperone complexes or extraction of soluble, misfolded protein from the ER and through ER-associated protein degradation (ERAD). The formation of insoluble protein aggregates requires clearance by autophagy. ER stress stimuli impair polypeptide folding and induce adaptive increases in chaperones and catalysts within the ER lumen through unfolded protein response (UPR) sensor activation. Chronic or overwhelming stimuli elicit a number of apoptotic signals including oxidative stress, cJun-N-terminal kinase (JNK) activation, CHOP expression, cleavage of caspase 12 and activation of the intrinsic mitochondrial-dependent cell death pathway. Physiological stimuli that can activate the UPR in the *β*-cell include expression of misfolded proinsulin or islet amyloid polypeptide (IAPP), oxidative stress [reactive oxygen species (ROS)] and increases in the extracellular concentrations of glucose, fatty acids or cytokines.

Protein misfolding occurs in the ER as a consequence of a number of insults, including pharmacological perturbation, alterations in calcium, redox status or nutrient availability, mutations in ER chaperones or their client proteins, viral infection, as well as differentiation of cells that secrete large amounts of proteins. The accumulation of unfolded proteins in the ER lumen activates the UPR [5],[10]. The UPR is an adaptive response designed to resolve the protein-folding defect by (i) reducing protein influx into the ER; (ii) increasing the capacity to promote productive protein folding and (iii) increasing the clearance of misfolded proteins in the ER lumen through ERAD and autophagy (figure 1). These strategies may exhibit temporally separate phases of the UPR. Reduced influx occurs rapidly and transiently, and prevents further generation of misfolded proteins, while upregulation of protein maturation and degradation machinery occurs after UPR-dependent gene induction. Over time, homeostasis may be re-established in the ER to resolve the protein-folding defect [11]. If homeostasis is not restored, the UPR is chronically activated and leads to apoptosis. The mechanisms that decipher the protein folding status and orchestrate a coordinated downstream response, either adaptation or apoptosis, are fundamentally significant, unknown, and require further investigation.

## UPR Translational and Transcriptional Control

**Figure 2:**
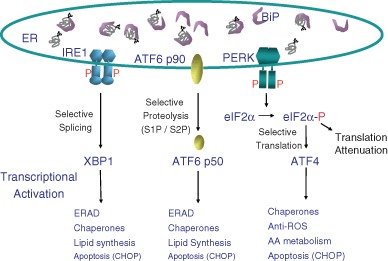
Signalling the unfolded protein response (UPR). The UPR sensors protein kinase-like endoplasmic reticulum kinase (PERK), inositol-requiring enzyme 1 (IRE1) and ATF6 control mRNA translation and transcriptional induction of UPR-regulated genes. Interaction of BiP with each UPR sensor prevents UPR signalling. Upon accumulation of unfolded protein, BiP is released from each sensor, leading to its activation. The ER protein kinase PERK is activated by homodimerization and autophosphorylation to phosphorylate eukaryotic initiation factor 2 *α*-subunit (eIF2*α*), thereby reducing the rate of mRNA translation and the biosynthetic protein-folding load on the ER. eIF2*α* phosphorylation paradoxically increases translation of *Atf4* mRNA to produce a transcription factor that activates expression of genes encoding protein chaperones, ER-associated protein degradation (ERAD) machinery, enzymes that reduce oxidative stress and functions in amino acid biosynthesis and transport. Dimerization of the ER protein kinase IRE1 triggers its endoribonuclease activity to induce cleavage of *Xbp1* mRNA. *Xbp1* mRNA is then ligated by an uncharacterized RNA ligase and translated to produce XBP1s. Concurrently, ATF6 released from BiP transits to the Golgi where it is cleaved to release a transcriptionally active fragment. Cleaved ATF6 acts in concert with XBP1s to induce expression of genes encoding protein chaperones and ERAD machinery. The RNase activity of IRE1 also degrades selective cellular mRNAs to reduce the client protein load upon the ER.

The UPR is signalled through activation of the protein kinases inositol-requiring enzyme 1 (IRE1) and protein kinase-like endoplasmic reticulum kinase (PERK) and cleavage of the transcription factor ATF6 (figure 2) [11],[12]. The three UPR sensors are maintained in an inactive state through interaction with the protein chaperone BiP. It is proposed that as unfolded proteins accumulate, bind and sequester BiP, they promote BiP dissociation from PERK, IRE1 and ATF6 [13],[14]. BiP release from IRE1 and PERK permits their homodimerization, trans-autophosphorylation and activation. Activated PERK phosphorylates the *α*-subunit of the translation initiation factor 2 (eIF2*α*) leading to rapid and transient inhibition of protein synthesis [15]. eIF2 is a heterotrimeric GTPase required to bring initiator methionyl tRNA to the ribosome for AUG initiation codon selection [16]. Phosphorylation of eIF2*α* inhibits the GDP/GTP exchange reaction on eIF2, thereby preventing eIF2 recycling and the initial step of polypeptide synthesis [15],[17],[18]. Paradoxically, eIF2*α* phosphorylation is required for the translation of several mRNAs (*Gadd34*, *Atf5*, *Cat1*), including the bZiP transcription factor ATF4 [19]. This mechanism apparently involves ribosomes scanning through upstream open reading frames in the 5′ end of the mRNA because of limiting amounts of GTP-bound eIF2-tRNA_met_.

Although there are *α*- and *β*-alleles of both IRE1 and ATF6 in the mammalian genome, only IRE1*α* is expressed in all tissues and only ATF6*α* signals the UPR. IRE1*β* is selectively expressed in intestinal epithelial cells and it is not known what genes are regulated by ATF6*β*. IRE1 activation elicits an endoribonuclease function that induces non-conventional splicing of *Xbp1* mRNA. Splicing of *Xbp1* mRNA, the only known splicing substrate of IRE1, removes a 26-base intron that alters the translation reading frame to produce a highly active bZiP transcription factor that activates genes encoding ER protein chaperones, lipid biosynthetic enzymes and ERAD functions [20]–[22]. Upon release from BiP, ATF6 traffics to the Golgi complex where it is cleaved by the S1P and S2P processing enzymes to produce a cytosolic fragment that activates transcription of genes providing complementary and overlapping functions with those activated by XBP1 which restores productive ER protein folding and increases ERAD [23]–[25]. Indeed, cells deleted in either *Ire1α*, *Xbp1 or Atf6α* are defective in ERAD [21],[25]–[27].

## Signals Downstream of eIF2*α* Phosphorylation – Regulation of Transcription by ATF4 and CHOP

During periods of ER stress, the selective translation of *Atf4* mRNA produces a factor that binds to the amino acid response element (AARE) in target genes [28],[29], such as *Atf3, Chop/Gadd153* and *Gadd34/MyD116/Ppp1r15a*[30],[31]. Recent studies, as well as our own, indicate chronic UPR activation causes induction of CHOP that is essential for the apoptotic response to chronic protein misfolding in the ER [32]–[34]. CHOP was originally isolated as a gene induced in response to DNA damage and it consists of a transcriptional activation/repression domain followed by a basic leucine zipper domain at its C-terminus [35]. Although it was thought that CHOP functions as a negative regulator to sequester binding partners [36], subsequent studies showed that a CHOP-C/EBP heterodimer could bind DNA and function as a transcriptional activator [37]–[39]. It is proposed that CHOP mediates induction of the apoptotic genes *Gadd34, Dr5, Bim* and *Trb3*, and repression of antiapoptotic *Bcl2* expression [33],[39],[40].

## Protein Misfolding in the ER and Oxidative Stress

There is accumulating evidence to suggest that protein misfolding in the ER and production of reactive oxygen species (ROS) are closely linked; however, this area of ER stress is not well explored. In eukaryotes, oxidative protein folding occurs in the ER. A growing family of ER oxidoreductases, including protein disulphide isomerase (PDI), ERp57, ERp72, PDIR, PDIp and P5, catalyse these protein-folding reactions in mammalian cells. PDI catalyses the formation, isomerization and reduction of disulphide bonds *in vitro*. When disulphide bond formation occurs, cysteine residues within the PDI active site [-C-X-X-C-] accept two electrons from thiol residues in the polypeptide chain substrate. This electron transfer results in the oxidation of the substrate and the reduction of the PDI active site. It is now recognized that reduced PDI transfers its electrons through ER oxidoreductase 1 (ERO1) to molecular oxygen as the final electron acceptor [41].

**Figure 3:**
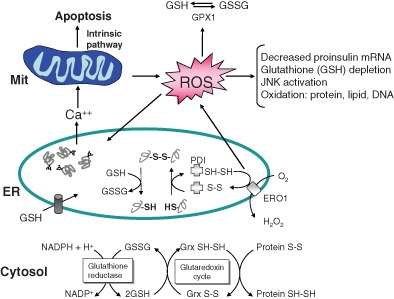
Endoplasmic reticulum (ER) stress, protein misfolding and oxidative stress are intimately interrelated. Protein folding within the ER lumen is ushered by a family of oxidoreductases that catalyse disulphide bond formation and isomerization. ER stress causes an increase in the formation of incorrect intermolecular and/or intramolecular disulphide bonds that require breakage and reformation for proteins to attain the appropriate folded conformation. Protein disulphide isomerase (PDI) catalyses disulphide bond formation and isomerization, whereas glutathione transported into the ER reduces improperly paired disulphide bonds. Reoxidation of PDI is mediated by ERO1; however, reactive oxygen species (ROS) are produced in the process. Cellular ROS can deplete glutathione and increase the misfolded protein load in the ER. In turn, ROS can also cause ER stress through modification of proteins and lipids that are necessary to maintain ER homeostasis. Consumption of excessive cellular glutathione due to ER stress could inhibit glutaredoxin reduction and cause accumulation of oxidized cytosolic proteins. ER stress also causes calcium leak from the ER for accumulation in the inner mitochondrial matrix. This calcium loading in the mitochondria can generate additional ROS through disruption of electron transport and opening of the mitochondrial permeability pore. Thus, accumulation of misfolded protein in the ER increases ROS production that can further amplify ER stress, disrupt insulin production and cause cell death.

During formation of disulphide bonds, hydrogen peroxide is formed as a by-product from the sequential action of PDI and ERO1 in transferring electrons from thiol groups in proteins to molecular oxygen. It has been estimated that approximately 25% of the ROS generated in a cell may result from formation of disulphide bonds in the ER during oxidative protein folding [41]. In addition, ROS may be formed as a consequence of the glutathione (GSH) depletion that occurs as GSH reduces unstable and improper disulphide bonds. The consumption of GSH would return thiols involved in non-native disulphide bonds to their reduced form so they may again interact with ERO1/PDI to be reoxidized. This would generate a futile cycle of disulphide bond formation and breakage in which each cycle would generate ROS and consume GSH (figure 3). As a consequence, it is expected that proteins that have multiple disulphide bonds may be more prone to generating oxidative stress. Our recent findings show that excessive protein synthesis and unfolded protein accumulation in the ER lumen can cause oxidative stress [42]. The ROS generated by these processes appear to be a critical second signal to initiate apoptosis (figure 3). In addition, our findings suggest that ROS can further disrupt protein misfolding in the ER. Intriguingly, antioxidants can improve protein folding and reduce apoptosis under conditions of ER stress.

## UPR Induction of ATF4 and CHOP and Apoptosis

**Figure 4:**
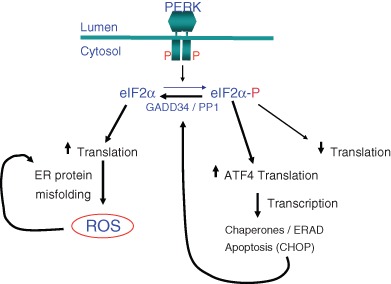
*β*-Cells require exquisite regulation of protein synthesis by eukaryotic initiation factor 2 *α*-subunit (eIF2*α*) phosphorylation. Our studies have shown that protein kinase-like endoplasmic reticulum kinase (PERK) activation initiates a cascade of events that include eIF2*α* phosphorylation, increased ATF4 mRNA translation and transcriptional activation of CHOP. Increased CHOP expression is associated with oxidative stress. However, we have also shown that a defect in eIF2*α* phosphorylation results in increased protein synthesis that also leads to oxidative stress. We propose that increased CHOP expression induces expression of GADD34 to direct protein phosphatase 1 (PP1)-mediated dephosphorylation of eIF2*α*, thereby increasing protein synthesis. If protein misfolding is not resolved, the increased cargo would further increase misfolded protein accumulation, leading to reactive oxygen species (ROS). The heavy black arrows depict the signalling pathway that occurs upon eIF2*α* phosphorylation.

How do cells decide between the fates of survival and death in response to activation of the UPR? Our recent studies, as well as others, indicate that CHOP is a crucial factor that signals apoptosis [43]. Surprisingly, cells in which the UPR is chronically activated at a low level can propagate indefinitely because the stress from the initial insult is resolved. The resolution correlates with upregulation of ER chaperones and ERAD machinery. Survival under these conditions is associated with attenuated expression of CHOP, and GADD34, a downstream target of CHOP [43]. In the presence of perpetual CHOP expression, cells succumb to apoptotic death. We showed that the half-lives for mRNAs and proteins encoding adaptive functions of the UPR (i.e. BiP, GRP94, p58^IPK^) are long-lived, whereas those encoding apoptotic functions, ATF4, CHOP and GADD34, are short-lived. Under adaptive conditions, the steady-state levels of proteins that promote protein folding and ERAD are increased, which feeds back to shut off UPR signalling. In contrast, a perpetual strong misfolded protein signal is required to activate the CHOP-mediated death program. We have shown that *Chop* deletion improves both *β*-cell survival and proinsulin secretion. Our studies suggest that CHOP mediates apoptosis through induction of oxidative stress. However, the relationship between signalling through the PERK/eIF2*α* pathway to production of ROS is unknown. A model was proposed that ATF4-mediated induction of CHOP causes transcriptional induction of GADD34 [31]. GADD34 encodes a regulatory subunit of protein phosphatase 1 (PP1) that targets PP1 to dephosphorylate eIF2*α*, thereby reversing the translational attenuation. We propose that under conditions where protein folding is challenged, an increase in protein synthesis would generate an increased amount of misfolded protein (figure 4). Although the most significant pathway that leads to CHOP induction is mediated through PERK, both the IRE1/XBP1 and ATF6 pathways contribute.

## UPR Activation in *β*-Cells Associated with Type 2 Diabetes

If ER stress and UPR signalling are important for *β*-cell function/survival, then (i) *UPR* gene induction should be detectable in the islets of diabetic mice and human patients; (ii) accumulation of unfolded protein in *β*-cells should cause *β*-cell failure and diabetes and (iii) genetic deletion of critical UPR signal transduction components should cause overwhelming ER stress and diabetes. There is now evidence to support each of these predictions.

### UPR Activation in Islets from Diabetic Mice and Men

Perhaps the most difficult test of UPR association with *β*-cell failure is the detection of ER stress or defective ER stress signalling in islet samples from human patients. However, CHOP nuclear localization was reported in pancreata of human obese diabetic individuals, but it was rarely found in perinuclear or nuclear localization in pancreata from control or type 1 diabetic patients [44]. The classical UPR-induced proteins p58^IPK^, BiP and CHOP were significantly elevated in islets in tissue sections from patients with type 2 diabetes [45]. Increased levels of eIF2*α* phosphorylation, increased splicing of *Xbp1* mRNA and increased CHOP and BiP protein were detected in the islets of *db/db* mice, a common model of insulin resistance and *β*-cell failure [45],[46]. The detection of UPR-induced signals in these samples does not prove that ER stress was a causative event in the disease process; however, it does provide the first evidence that UPR markers are elevated specifically in the islets of diabetic men and mice. Further substantiating that excessive ER stress or defective stress signalling are pathogenic determinants of human diabetes will require more advanced knowledge of the stimuli and function of the UPR in *β*-cells and the development of sensitive methods to detect markers of the UPR in human samples. One outstanding question is whether insulin resistance causes an increase in proinsulin synthesis that generates greater amounts of misfolded protein in *β*-cells. The discovery of drugs that improve ER protein folding and/or modulate ER stress signalling, which can be evaluated in diabetic animal models and in human clinical trials, will greatly advance our understanding of the importance of ER stress in development and progression of diabetes.

### *β*-Cell Failure: Mutant Proinsulin

Studies of *Akita* and *Munich* mice show that mutations at cysteine residues that interfere with proper disulphide bond formation within proinsulin induce ER stress and severe *β*-cell destruction [47],[48]. Deletion of the UPR-induced proapoptotic gene *Chop* delayed onset of hyperglycaemia and *β*-cell failure in the *Akita* mouse [48]. Human proinsulin with the analogous *Akita C*96*Y* mutation was analysed and compared with wild-type proinsulin through the development of expression constructs that fuse green fluorescence protein (GFP) with the C-peptide [49]. In these studies, it was possible to elucidate that processing of hPro*C*96*Y* CpepGFP to insulin was completely impaired in rat insulinoma-1 (INS-1) cells and expression was ‘proteotoxic’ in comparison to control hProCpepGFP. In humans, neonatal dominantly inherited diabetes in 16 families was associated with missense mutations in the *Ins* gene [50]. The mutations are predicted to impair proinsulin disulphide bond formation and activate ER stress. The missense mutations affect residues directly involved in disulphide bond formation, crucial residues adjacent to disulphide bridges, and also introduce new cysteine residues that could interfere with correct pairing of cysteine residues as nascent proinsulin molecules undergo oxidative folding. Thus, disruption of disulphide bond pairing in proinsulin, a crucial determinant of secondary structure and protein folding, is sufficient to induce diabetes in both humans and mice.

### *β*-Cell Failure: Deletion of ER Co-chaperone p58^IPK^

p58^IPK^ was first described as an inhibitor of the double-stranded RNA-activated eIF2*α* protein kinase R (PKR). It was subsequently shown to inhibit activation of the eIF2*α* kinase PERK [51],[52]. The subcellular localization and function of this protein has been a subject of debate; however, recent evidence supports the notion that the majority of p58^IPK^ is imported into the ER lumen [53]. p58^IPK^ is a member of the DnaJ co-chaperone family that functions to stimulate the ATPase activity of members of the Hsp70 family. Therefore, it was proposed that p58^IPK^ may act in the ER lumen as a co-chaperone for the Hsp70 family member BiP [53]. Mice with null mutation of *p*58^IPK^ develop spontaneous diabetes because of destruction of the islet mass, and **************p58^IPK^ null mutation worsens the outcome of diabetes due to the *Akita Ins2 C96Y* mutation [54],[55]. These intriguing findings merit further study on the role of p58^IPK^ co-chaperone function in proinsulin folding and maturation and in diabetes. The observations suggest that there may be a number of protein-folding chaperones that play highly significant roles in preservation of ER function in the *β*-cell to prevent diabetes.

### *β*-Cell Failure: Wolfram Syndrome and ER Dysfunction

Wolfram syndrome is a rare autosomal-recessive neurodegenerative disorder that is characterized by juvenile-onset diabetes mellitus, optic atrophy and hearing impairment [56]. This syndrome is caused by loss-of-function mutations in the *Wfs1* gene that encodes the protein wolframin [57],[58]. Although WFS1 is not a direct sensor of the UPR, analysis of *Wfs1*−*/*− mice indicates that WFS1 function is closely linked with ER homeostasis. *Wfs1* null mutation reduces intracellular calcium signalling upon glucose stimulation, induces UPR-regulated genes and disrupts cell cycle control, leading to apoptosis [59]–[61]. Recently, a physical interaction between WFS1 and the Na(+)/K(+)ATPase *β*1 subunit was discovered, and it was discerned that WFS1 was required for trafficking of the subunit to the cell surface. Reduced levels of this ATPase subunit were detected in the plasma membrane fraction of *Wfs1* mutant fibroblasts and of *Wfs1* knockdown MIN6 *β*-cells [62]. Wolframin may serve a general function to assist in the assembly of subunits of oligomeric proteins before exit from the ER. Consistent with these observations, loss of function in the chaperone WFS1 causes ER stress and *β*-cell failure.

### *β*-Cell Failure: Mutations in PERK/eIF2*α*

Wolcott-Rallison syndrome was first reported in the early 1970s as a human disease characterized by infantile diabetes, multiple epiphyseal dysplasia and growth retardation [63],[64]. Pancreas atrophy and endocrine and exocrine insufficiency were observed [65],[66]. Wolcott-Rallison syndrome has been associated with multiple other pathologies including osteopenia, hepatic and renal complications, cardiovascular disease and mental retardation. Remarkably, it was learned nearly 30 years later that this syndrome results from loss of protein kinase function mutations in the eIF2*α* kinase PERK (EIF2AK3) [67],[68]. Furthermore, polymorphisms at the *PERK* locus were linked to type 1 diabetes in South Indian populations [69].

*Perk* deletion in the mouse recapitulates many of the defects of the human syndrome including diabetes due to degeneration of *β*-cell mass after birth and failure of the exocrine pancreas [70],[71]. In these studies, ER distention, a characteristic of ER dysfunction, was observed in pancreatic *β*-cells. In addition, the rate of glucose-stimulated proinsulin synthesis was enhanced, consistent with a defect in the ability to properly attenuate proinsulin mRNA translation. The findings suggested that the *β*-cells of these mice were susceptible to ER overload and unresolved ER stress leading to apoptosis. Conditional deletion of *Perk* at varying times in development suggested that the development of *β*-cell mass, but not maintenance of a population of adult *β*-cells, is dependent upon this kinase [72]. It is possible that one or more additional eIF2*α* kinases, general amino acid control 2 (GCN2), heme-regulated inhibitor (HRI) or ds-RNA-activated protein kinase (PKR), are capable of supporting the minimal requirement for eIF2*α* phosphorylation and translational control in response to *in vivo* stimuli.

Concurrent with studies on *Perk* null mice, mice that harbour a homozygous knock-in mutation at the PERK phosphorylation site in eIF2*α* (*Ser51Ala*) were shown to have defects in embryonic *β*-cell survival, liver glycogen storage, postnatal induction of gluconeogenesis, inhibition of translation under conditions of ER stress and transcriptional induction of *UPR* genes [17]. The *Ser51Ala eIF2α* mutation prevents any compensatory phosphorylation because of activation of other eIF2*α* kinases and therefore, very effectively blocks stress-induced translation attenuation and ATF4-dependent transcriptional induction. Mice with homozygous *Ser51Ala eIF2α* mutation die from postnatal hypoglycaemia. This observation was the first indication that the UPR has a broader role than maintaining functional ER protein folding, but is also intimately connected with metabolic homeostasis [17]. It is now known that all UPR signalling pathways contribute to glucose and lipid homeostasis [22],[73],[74]. Because the homozygous *Ser51Ala eIF2α* mutation was a neonatal lethal phenotype with a severe *β*-cell deficiency, further studies were performed by analysis of *β*-cell function in heterozygous *Ser51Ala eIF2α* mice [75]. The heterozygous animals did not spontaneously manifest *β*-cell failure because of reduced ER stress signalling. However, upon feeding a 45% high-fat diet, these mice developed elevated fasting blood glucose, glucose intolerance and a *β*-cell secretion defect. It was shown that the insulin secretion defect was because of an increased rate of glucose-stimulated translation that overwhelmed the protein folding machinery of the ER and led to (i) distention of the ER compartment, (ii) prolonged association of misfolded proinsulin with the ER chaperone BiP and (iii) reduced secretory granule content. Thus, the regulation of translational initiation through eIF2*α* phosphorylation is required for ER stress signalling to prevent *β*-cell dysfunction, when insulin demand is increased because of a high-fat diet and insulin resistance. These findings showed that translational control through eIF2*α* phosphorylation is essential to maintain the functional integrity of the ER.

As ER distention and *β*-cell death in homozygous *Ser51Ala eIF2α* islets were apparent embryonically in the absence of any exogenous pressure to drive *β*-cell failure, it is likely that there are physiological stimuli that cause eIF2*α* phosphorylation early in development that is crucial for *β*-cell survival [17]. It is unlikely that the *β*-cell requirement for eIF2*α* phosphorylation is mediated through increased translation of *Atf4* mRNA, because *Atf4*−/− mice do not display *β*-cell defects [76]. However, the embryonic *β*-cell apoptosis observed in homozygous *Ser51Ala eIF2α* mice was likely, at least in part, signalled through CHOP. Although disruption of the *Chop* gene did not rescue the postnatal hypoglycaemia-induced lethality of *Ser51Ala eIF2α* mice, the *β*-cells in islets from E18.5 *Ser51Ala eIF2α*;*Chop*−/− mice were significantly increased in number and insulin content, and displayed reduced apoptosis compared with islets from *Ser51Ala eIF2α*;*Chop*+*/*+ mice [77]. These findings support the hypothesis that a significant portion of the *β*-cell apoptosis in *Ser51Ala eIF2α* mutant mice is caused by CHOP, and that CHOP induction is not solely dependent on eIF2*α* phosphorylation. However, importantly, these findings show that CHOP is not the only pathway leading to *β*-cell death in the absence of eIF2*α* phosphorylation. In the absence of CHOP, other death pathways involving both mitochondrial-dependent and -independent pathways are invoked. The sum of these findings indicates that genetic defects in the PERK/eIF2*α* signal transduction pathway are sufficient to disrupt regulated mRNA translation and interfere with ER function in the *β*-cell, thereby causing reduced insulin secretion, *β*-cell death and diabetes in mice and humans.

## The Role of Protein Misfolding and Oxidative Stress in *β*-Cell Failure

To determine whether CHOP contributes to the *β*-cell failure associated with type 2 diabetes, we analysed the effect of *Chop* deletion in three different models of murine type 2 diabetes: (i) heterozygous *Ser51Ala eIF2α* mutant mice fed a high-fat diet; (ii) high-fat diet and streptozotocin (STZ)-treated mice and (iii) *db/db* leptin receptor-null mice [77]. In all models, *Chop* deletion increased *β*-cell mass, improved *β*-cell function, reduced *β*-cell apoptosis and prevented glucose intolerance. Surprisingly, *Chop* deletion not only reduced apoptosis but also preserved *β*-cell function, as monitored by the integrity of the secretory pathway, insulin expression and glucose-stimulated insulin secretion. Analysis of gene expression suggested that *Chop* deletion may improve the capacity of the *β*-cell to tolerate oxidative stress. Indeed, islets from *Chop*−/− mice displayed no significant difference in oxidative damage compared with wild-type mice. However, upon incubation in the presence of tunicamycin to inhibit *N*-linked glycosylation and cause misfolded protein accumulation in the ER, there was a significant increase in protein oxidation and lipid peroxidation in wild-type islets, where the damage was significantly attenuated in *Chop*−/− islets [77]. In contrast, upon treatment with the oxidant H_2_O_2_, similar amounts of oxidative damage were detected in islets from both strains of mice. Therefore, *Chop* deletion reduced the oxidative damage that occurs in response to protein misfolding in the ER, but not in response to general oxidative stress. We conclude that *Chop* deletion not only promoted islet hyperplasia but surprisingly also improved the function of the *β*-cells to maintain insulin production, possibly through reduction of oxidative stress.

## eIF2*α* Phosphorylation Prevents Oxidative Stress in *β*-Cells

To elucidate why eIF2*α* phosphorylation is required to preserve *β*-cell function, we generated mice with conditional homozygous *Ser51Ala eIF2α* mutation in *β*-cells. Ubiquitous transgenic expression of wild-type *eIF2α* cDNA rescued the postnatal lethality associated with the homozygous *Ser51Ala eIF2α* mutation. The rescued mice were viable, fertile and displayed normal glucose tolerance and homeostasis. Locus of X-over P1 (LoxP) sites flanked the wild-type *eIF2α* cDNA within the transgene so that when deleted, GFP was expressed. Upon breeding these mice to transgenic mice harbouring the rat insulin II promoter tamoxifen (Tam)-regulated Cre recombinase [78], the wild-type *eIF2α* cDNA was efficiently deleted in more than 90% of the *β*-cells. Three weeks after administration of Tam, nearly all of the *β*-cells had deleted the wild-type *eIF2α* cDNA, although there was no significant change in islet mass or insulin content and the mice were glucose intolerant [76]. At 3 weeks after Tam injection, there were terminal deoxynucleotidyl transferase mediated dUTP nick end labeling essay (TUNEL)-positive *β*-cells, prior to the onset of detectable hyperglycaemia, suggesting that *β*-cell failure and apoptosis were not a consequence of lipid or glucose toxicity [76]. In addition, ultrastructural analysis identified significantly distended ER and swollen mitochondria in *β*-cells at 3 weeks after deletion of the transgene. Finally, oxidative damage was detected co-incident with the appearance of TUNEL-positive *β*-cells, suggesting that blockade of ER stress signalling by preventing eIF2*α* phosphorylation overloads the ER compartment causing accumulation of unfolded protein, oxidative stress and subsequent irreversible commitment to cell death.

We explored the causal relationship between ROS and disruption of glucose homeostasis in this *in vivo* mouse model of diabetes due to translation-induced overload. We found that diet supplemented with antioxidant butylated hydroxyanisole (BHA) could reduce *β*-cell apoptosis, increase insulin content and improve glucose homeostasis in mice with homozygous *Ser51Ala eIF2α* mutation in *β*-cells [76]. These findings suggest that reducing ROS in *β*-cells deficient in eIF2*α* phosphorylation could improve their function and that ROS may be a cause for the *β*-cell failure and apoptosis.

## The Role of IRE1*α* and ATF6*α* UPR Signalling in *β*-Cells

A fundamental question regarding *β*-cell function and survival is which UPR subpathways are required for *β*-cell function and what elements of these responses are protective or detrimental to *β*-cell survival upon acute or unresolved ER stress. We have recently shown that *β*-cell-specific deletion of *Ire1α* causes hyperglycaemia because of *β*-cell failure (unpublished). Therefore, signalling through IRE1*α* is apparently also required to preserve *β*-cell function. In contrast, mice with homozygous deletion of *Atf6α* displayed normal glucose homeostasis on a standard chow diet. Future studies should investigate whether high-fat diet may uncover a requirement for ATF6*α* for *β*-cell compensation when challenged with insulin resistance. Evidence that defects in the IRE1*α* and ATF6*α* subpathways of the UPR are akin to null mutation of PERK in causing human diabetes has yet to be presented. However, further analysis of mouse models with *UPR* gene deletions should solidify the concept that the UPR sensors act in concert with each pathway, supporting a unique and indispensable role in preservation of *β*-cell function and/or survival.

## Conclusion

Our results show that the absence of eIF2*α* phosphorylation, as well as excessive eIF2*α* phosphorylation (through CHOP induction), leads to oxidative stress. We propose that both events lead to increases in protein synthesis that contributes to the oxidative stress (figure 4). Presently, most data support the idea that CHOP is induced by the PERK/eIF2*α*/ATF4 as well as the IRE1/XBP1 and ATF6 UPR subpathways to activate proapoptotic gene expression, restore translation initiation and increase the oxidizing potential in the ER lumen [31],[79]. CHOP induces expression of GADD34, a subunit of type 1 protein phosphatase that directs eIF2*α* dephosphorylation to increase mRNA translation as homeostasis in the ER is restored [31],[80]. CHOP is also implicated in the induction of ERO1*α*, a molecule that oxidizes PDI so it can function to rearrange improperly formed disulphide bonds within unfolded proteins. Disulphide bond formation during oxidative protein folding in the ER generates oxidative stress as a consequence of electron transfer from cysteine residues through PDI and ERO1 to molecular oxygen to form hydrogen peroxide [41]. Future studies should elucidate whether *Chop* deletion protects *β*-cells from oxidative damage through the reduced expression of GADD34 and/or ERO1.
